# Physicochemical Properties and Consumer Acceptance of Yak Mozzarella Cheese Produced by Culture Acidification and Direct Acidification

**DOI:** 10.3390/foods15020252

**Published:** 2026-01-10

**Authors:** Puwei Yan, Lan Mi, Li Song, Yingrui Lu, Qi Liang, Liya Zhang, Yan Zhang, Yinhua Zhu

**Affiliations:** 1 College of Food Science and Engineering, Gansu Agricultural University, Lanzhou 730070, China; yanpw@st.gsau.edu.cn (P.Y.);; 2 Gansu Hualing Dairy Co., Ltd., Hezuo 747000, China; 3 Gansu Bozhenyuan Biological Dairy Co., Ltd., Wuwei 733099, China; 4 Department of Nutrition and Health, China Agricultural University, Beijing 100083, China

**Keywords:** yak milk, mozzarella cheese, acidification, proteolysis, consumer acceptance

## Abstract

The rate of acidification (pH = 6.1) at an appropriate degree is responsible for supplying suitable aroma components and functional properties to cheese. This study aimed to evaluate variations in physicochemical, functional properties, and consumer acceptance in yak mozzarella cheese produced using different starter cultures or lactic acid during ripening. The results showed that consumers preferred ripened yak M cheese, made with mesophilic multi-strain starter, which received the highest scores for aroma (6.8) and flavor (5.9). The average levels of most major volatile organic compounds were relatively higher in ripened M cheese. Furthermore, the degree of proteolysis increased continuously during the 42 d ripening period. The contents of pH 4.6-soluble nitrogen and 12% trichloroacetic acid-soluble nitrogen in cheeses produced with starter cultures reached 11% and 8%, significantly higher than those of directly acidified L cheese. Specifically, greater protein degradation corresponded to lower hardness and stretchability, hardness of T and M cheeses decreased from 226.67 ± 2.23 g and 232.87 ± 3.66 g to 202.36 ± 2.63 g and 197.09 ± 2.33 g, respectively, while their stretchability declined from 52.1 ± 1.6 cm and 49.3 ± 1.7 cm to 34.5 ± 1.2 cm and 37.6 ± 2.4 cm. However, yield and moisture content of T and M cheeses were significantly lower than those of L cheese. Overall, this study provides valuable insights for optimizing the production and quality of yak mozzarella cheese.

## 1. Introduction

Yak (*Bos grunniens*) milk is a key economic resource on the Qinghai–Tibet Plateau, with an annual output exceeding 1.2 million tons. Known as a naturally concentrated milk, it contains higher levels of dry matter (~17 g/100 mL), protein (~5 g/100 mL), and fat (~6 g/100 mL) than cow milk [1,2]. However, most yak milk is still processed into low-value Qula, resulting in limited product diversification [3,4]. Mozzarella cheese dominates China’s imported cheese market, accounting for over 50% of the market share [5]. Therefore, yak milk represents a promising alternative for cheese making. Its higher calcium phosphate content and larger casein micelles yield stronger rennet-induced gels and higher cheese yields (20.53%) than cow milk [6]. However, the impact of acidification strategy (starter-based fermentation versus direct acidification) and starter type (thermophilic versus mesophilic) on key quality attributes—such as proteolysis, texture, meltability, stretchability, and the formation of volatile flavor compounds—has not been systematically examined for yak Mozzarella. Therefore, this study aimed to address this gap by testing the following hypotheses: (1) Starter-fermented cheeses will develop more complex volatile profiles and better texture–function balance than directly acidified cheese; (2) Thermophilic and mesophilic starters will produce distinct proteolytic and flavor outcomes due to differences in enzyme activity and acidification kinetics; (3) Direct acidification, despite its higher moisture and calcium retention, will lead to inferior flavor and lower consumer preference.

Acidification, the “heart of cheesemaking” [7], determines the rate and extent of acid development crucial for mozzarella production. Both starter cultures and direct acidification can be used [8]. Direct acidification with citric or lactic acid shortens processing time [9], whereas starter culture fermentation produces firmer curds with higher water retention [10]. Acidification enhances solubilization of colloidal calcium phosphate and calcium release [11]. Lactic acid bacteria (LAB) further enrich flavor, texture, and nutritional value by releasing bioactive metabolites [12,13]. Yet, suitable starter cultures or acidification methods for yak mozzarella remain unexplored.

Mozzarella can be made with mesophilic or thermophilic starters, depending on processing temperature [14]. The primary acid producers are *Lactococcus lactis* subsp. *lactis*, *Lactococcus lactis* subsp. *cremoris*, *Streptococcus thermophilus*, and *Lactobacillus delbrueckii* subsp. *bulgaricus*. *Streptococcus thermophilus* produces formic acid that stimulates *Lactobacillus delbrueckii* subsp. *bulgaricus* growth [15], while lactobacilli contribute strong proteolytic activity and flavor formation [16]. While the formic acid-mediated mutualism between *Streptococcus thermophilus* and *Lactobacillus delbrueckii* subsp. *bulgaricus* is best characterized in yogurt; this interactive dynamic remains relevant when these species are co-cultured in the cheese. Recently, *Lactococcus lactis* subsp. *cremoris* and *Lactococcus lactis* subsp. *lactis* has been used jointly as a commercial starter due to its complementary properties: rapid acidification by *L. lactis* subsp. *lactis* and enhanced curd viscosity by *L. lactis* subsp. *Cremoris* [17]. As yak milk composition differs from cow milk, microbial metabolism and flavor compound development may also vary, necessitating optimization of acidification rate and starter selection.

Developing high-quality yak mozzarella is vital for dairy industrialization on the Qinghai–Tibet Plateau. Using commercial starter cultures or direct acidification offers an efficient and economical strategy. Therefore, this study compared the physicochemical properties, texture, and consumer acceptance of yak mozzarella cheeses produced by culture acidification (using thermophilic (Danisco China Co., Ltd., Suzhou, China) and mesophilic multi-strain starters (Danisco China Co., Ltd., Suzhou, China)) and direct lactic acid acidification.

## 2. Materials and Methods

### 2.1. Yak Mozzarella Cheese Preparation

Yak milk used in this study was obtained in August from Zhuxixiulong Town in Tianzhu, Qinghai–Tibetan Plateau, and brought to the laboratory under refrigerated conditions. Raw milk was standardized, and the protein content was 4.8 g/100 g, the protein-to-fat ratio was 1.2.

Thermophilic multiple strain starter YF-L904 (*Lactobacillus delbrueckii* subsp. *bulgaricus* and *Streptococcus thermophilus*, TS) and mesophilic multiple strain starter R-704 (*Lactococcus lactis* subsp. *cremoris* and *Lactococcus lactis* subsp. *Lactis*, MS) were used as starter cultures (Danisco China Co., Ltd., Suzhou, China). Inoculation rate of 0.5% (*w*/*v*). Powdered calf rennet (chymosin:pepsin = 7:3; activity: 890 IMCU/g; Beijing Duoaite Bio-Technology Co., Ltd., Beijing, China) was used as the coagulating enzyme.

Yak Mozzarella cheese was produced in three independent batches per treatment following a modified method based on Akarca [18]. In brief, standardized yak milk was pasteurized at 63 °C for 30 min. Following pasteurization, the milk was actively cooled to 35 °C within 20 min under gentle agitation (60 rpm). The cooled milk was divided into 1 L aliquots and subjected to three treatments: addition of thermophilic starter (TS) at 0.618 g/L, mesophilic starter (MS) at 0.522 g/L, or a lactic acid solution (85%, *w*/*w*). The lactic acid solution was diluted tenfold with sterile water and added dropwise under continuous stirring over 2 min to immediately achieve a pH of 6.1 (±0.05). For the TS and MS cheeses, the respective starters were added to the milk and incubated at 35 °C for 40 min until the pH reached 6.1. Throughout the acidification process, pH was monitored intermittently at 5-min intervals using a calibrated pH meter. After acidification, coagulation was initiated with CaCl_2_ (0.03%, *w*/*w*) and rennet (1.2%, *w*/*w*). After reaching the coagulation point at approximately 30 min (flocculation time), the curd was cut into 1 cm^3^ cubes. The curd particles were then gently stirred (15 rpm) while being heated from 35 °C to 42 °C over 20 min and held at 42 °C for 15 min. Thereafter, the curds underwent a cheddaring process with turning every 15 min for about 2 h until the pH dropped to 5.2–5.3. The curd was then milled, mixed thoroughly with dry salt (NaCl, 8%, *w*/*w*), and manually stretched in hot water at 80 °C (water-to-curd ratio 2:1; internal curd temperature 58–60 °C) for 5 min. After shaping, the cheese was immersed in brine (NaCl 20 g/L, 10 °C, brine-to-cheese ratio 3:1) for 1 h, yielding a final salt content of approximately 1.2% (*w*/*w*). The vacuum-packaged cheeses were stored at 4 °C. Sampling was performed within 24 h post-manufacturing (defined as day 0) and subsequently on days 7, 14, 21, 28, 35, and 42, with triplicate samples taken from three independent production batches per treatment group.

The cheese prepared with TS and MS is referred to as T and M cheese, respectively. And the L cheese is referred to that manufactured by direct acidification method with addition of lactic acid.

### 2.2. Physicochemical Characteristics and Proteolysis Determination

The basic composition of cheese samples at various ripening stages was determined in accordance with Chinese National Standards. Moisture content was measured by the direct drying method (GB 5009.3-2016 [19]). Protein content was determined using the Kjeldahl method (GB 5009.5-2016 [20]). Calcium content was determined by flame atomic absorption spectrometry (GB 5009.92-2016 [21]). The pH of each cheese was measured using pH meter (S400-K, Mettler Toledo Technology (China) Co., Ltd., Shanghai, China) according to To [22] by mixing 5 g of the mozzarella cheese sample and 10 mL distilled water.

Moreover, pH 4.6-SN and 12% TCA-SN were determined according to To [22]. 0.75 g of grated cheese was homogenized with 50 mL of acetate buffer (0.2 M, pH 4.6). The homogenate was centrifuged at 6000× *g* for 20 min at 4 °C, and the supernatant was collected. For the 12% TCA-SN, 1.5 g of grated cheese was treated with 50 mL of 12% (*w*/*w*) TCA aqueous solution and processed using the same homogenization and centrifugation procedure. The nitrogen content in both supernatants was determined using the Kjeldahl method, and the results were expressed as a percentage of the total nitrogen content in the same cheese sample.

### 2.3. Cheese Functional Properties and Texture Profile Analysis (TPA)

#### 2.3.1. Stretchability

Cylindrical cheese samples (2 cm diameter × height) were equilibrated at room temperature for 30 min and then heated at 200 °C for 5 min. The melted curd was immediately stretched vertically upward by the same operator at a consistent speed using a fork until the strand broke. The pre-break length was recorded. Tests were performed in triplicate per sample, with results reported as mean ± standard deviation.

#### 2.3.2. Meltability

The meltability of the yak mozzarella cheeses was determined using a modified Schreiber melt test [23]. Cylindrical cheese samples with a diameter of 18 mm and a thickness of 7 mm were taken and balanced for 30 min at room temperature. Each sample was then placed in a sealed, heat-resistant glass beaker to minimize moisture evaporation and heated in an oven at 100 °C for 1 h. After molten curds were cooled at room temperature for 30 min, the vertical diameter was measured. The meltability was defined as the average value of two diameter readings. The tests were replicated three times.

#### 2.3.3. TPA

The texture of yak Mozzarella cheese was analyzed using a TA.XT Plus texture analyzer (Stable Micro Systems, Godalming, UK) fitted with a P25 probe and a 25 kg load cell. Cylindrical samples (2.0 cm diameter × height) were equilibrated at 25 ± 1 °C for 30 min and then subjected to a two-bite TPA test (50% deformation, 1.0 mm/s test speed, 5.0 mm/s pre-/post-test speed, 5 g trigger force, 5 s between compressions). Each sample was measured in triplicate.

### 2.4. Volatile Organic Compounds (VOC) Analysis and Consumer Acceptance Test

The VOC was extracted by headspace solid-phase microextraction (SPME) and subsequently separated and identified by GC-MS according to Natrella [24] with some modification. 20 g of yak mozzarella sample was minced with 10 g of anhydrous sodium sulfate, 1 g of sodium chloride, and 10 µL of a 100 µg/mL 4-methyl-2-pentanone internal standard, and placed in a 50 mL glass vial. After equilibration at 30 °C for 15 min under continuous magnetic stirring, a SPME fibre (75 μm CAR/PDMS) was inserted to adsorb VOC from the vial headspace for 30 min (under continued agitation). Then, VOCs were desorbed at 250 °C for 5 min in the injection port operating in splitless mode (2 min). The molecules were analyzed by GC-MS system (6890/5973, Agilent Technologies, Santa Clara, CA, USA) equipped with a Quartz capillary column (30 m × 0.25 mm, 0.50 μm). The analysis was performed under the following conditions: oven temperature, 50 °C for 1 min, then increased at 5 °C/min to 220 °C and held for 10 min; helium carrier gas constant flow, 1.0 mL/min; injection in splitless mode (split valve closed for 2.0 min). The SPME fiber was desorbed in the injector (liner) maintained at 250 °C for 5.0 min. The mass detector was set under the following conditions: electron ionization (EI) ion source at 70 eV; ion source temperature 230 °C; quadrupole temperature 150 °C; transfer line temperature 280 °C; mass scan range 15–550 *m*/*z*.

Tentative identification of compounds was based on the comparison of their retention times with those of authentic standards and by matching their mass spectra against the reference spectra in the National Institute of Standards and Technology (NIST) library. Quantification was achieved using a multi-point calibration curve, and results are expressed in µg/kg.

Consumer acceptance testing was analyzed to determine consumer preferences for the flavor and texture of yak mozzarella cheese. The protocol used for data collection complied with the Declaration of Helsinki and the national ethical requirements. All panelists provided informed consent and no coercion to participate, and the collected data was anonymous as no personally identifiable information was used. Self-reported mozzarella cheese shoppers who purchased mozzarella cheese at least three times a year (*n* = 53; 68% female, 32% male; mean age 42 ± 12 years) were recruited. All cheese samples, including the conventional control, were stored at 4 °C and tempered to 10 °C before serving. Sample presentation order was randomized and balanced across panelists using a Williams design to control for order and carryover effects. Samples were cut into 3 × 3 cm cubes and presented in square containers labeled with random three-digit blinding codes. Consumers rated their liking of aroma, appearance, color, flavor, texture, and creaminess using a 9-point hedonic scale (1 = dislike extremely, 9 = like extremely). Attribute intensity was evaluated on a 5-point just-about-right (JAR) scale (1 = much too weak/soft, 3 = JAR, 5 = much too strong/hard). Purchase intent was measured with the question: “Based on the sample you just tasted, how likely are you to purchase this yak mozzarella cheese instead of your usual brand?” (5-point scale: 1 = very unlikely, 5 = very likely). A JAR penalty analysis (mean drop in overall liking) was performed to identify attributes driving disliking. Spring water was provided, and a 3-min enforced break was implemented between samples.

### 2.5. Statistical Analysis

Statistical analysis was conducted using SPSS 19.0 (IBM Corp., Armonk, NY, USA). For parameters measured across storage times, a repeated-measures ANOVA (mixed model) was applied with treatment, storage time, and their interaction as fixed factors. Significant effects (*p* < 0.05) were further examined using Duncan’s multiple range test. All samples were analyzed in duplicate or triplicate as technical replicates.

## 3. Results

### 3.1. Composition of Yak Mozzarella Cheese

**Figure 1:**
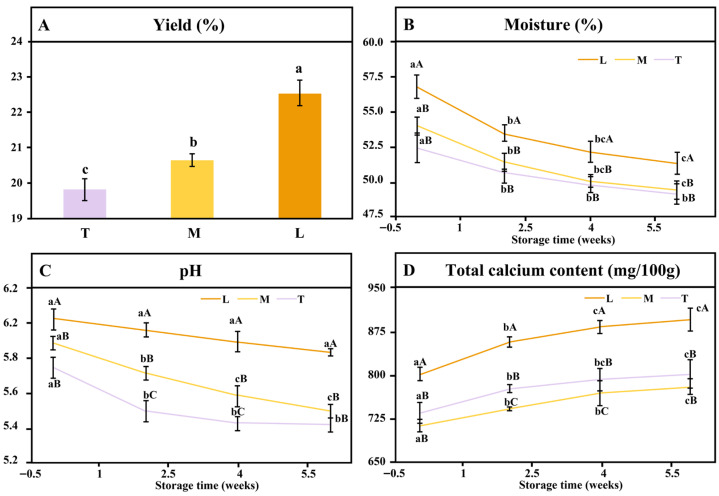
Gross composition and yield of yak mozzarella cheese ((**A**) the yield of yak mozzarella cheeses; (**B**) the moisture of yak mozzarella cheeses; (**C**) the pH of yak mozzarella cheeses; (**D**) total calcium content of yak mozzarella cheeses). Different lowercase letters indicate that the values of a parameter within the same group are significantly different after a specific storage duration, whereas different uppercase letters indicate that the values of a parameter within the same group are significantly different at the same storage time (*p* < 0.05). Error bars represent standard deviation.

The yield and physicochemical characteristics of yak mozzarella cheeses produced using starter cultures or direct acidification are shown in Figure 1. Significant differences were observed in yield, pH, moisture, and calcium content between cheeses made by culture acidification and direct acidification. The yield of yak milk cheese, generally higher than that of Holstein or Jersey milk [25], was lowest in T and M cheeses but highest in L cheese produced by direct acidification (Figure 1A). The moisture content was highest in the L cheese and lowest in the T cheese. This divergence can be attributed to their distinct acidification mechanisms. In the L cheese, rapid direct acidification promoted the dissolution of colloidal calcium phosphate, releasing a greater amount of calcium ions that contributed to the formation of a dense, water-retentive, yet less elastic protein network [26,27]. This structure limited the contraction of the curd during subsequent processing, resulting in lower whey expulsion efficiency and consequently higher final moisture content [28]. In contrast, the T cheese underwent slow biological acidification via starter culture, leading to a more gradual release of calcium ions and the development of a looser, more elastic protein matrix. This network facilitated curd shrinkage and efficient whey drainage during cooking and stirring, thus yielding a lower moisture content. During ripening, the moisture content of all cheeses decreased significantly, especially within the first two weeks. The L cheese exhibited the greatest absolute moisture loss, likely due to its higher initial moisture content—providing a larger absolute amount of water available for evaporation and biochemical reactions—combined with its denser matrix, which may have restricted uniform internal moisture migration and thereby accentuated the macroscopic reduction in moisture (Figure 1B).

pH values varied significantly among samples (*p* < 0.05). Fresh L cheese had a pH of 6.02 ± 0.06, similar to directly acidified cow mozzarella [9]. Throughout ripening, the fermented cheeses (T and M) exhibited lower pH values and showed a gradual decline in pH due to ongoing organic acid production [29]. Interestingly, L cheese exhibited no post-acidification, indicating pH stability during storage. In contrast, M and T cheeses showed significant pH reduction within the first three weeks. The acidifying profile of lactic acid bacteria is crucial for cheese technology [1]. *S. thermophilus* acidifies more rapidly and tolerates lower pH than *L. lactis* [30,31], explaining the lower pH of T cheese compared to M cheese after two weeks. The restricted post-acidification in T cheese likely stems from the inhibition of lactose metabolism in *L. bulgaricus* and *S. thermophilus* during storage at 4 °C, which consequently reduces their acid production [32].

Calcium dynamics also depended on the acidification method. Starter-based acidification to pH 6.1 caused higher calcium losses than lactic acid acidification (Figure 1D). Acid addition alters micellar calcium equilibrium depending on acid type and pH [33]. Pre-rennet acidification enhances calcium solubilization, but excessively rapid acidification limits colloidal calcium release [18,24]. In L cheese, the rapid addition of concentrated lactic acid may have induced micellar flocculation and restricted the diffusion of intramicellar calcium [34]. In contrast, the gradual pH decrease driven by starter cultures likely promoted more progressive and stable calcium solubilization. During ripening, the calcium content expressed on a dry-matter basis increased by approximately 10% in all cheeses, consistent with reports that colloidal calcium stabilizes during storage [35,36]. This increased concentration is likely due to moisture loss.

### 3.2. Proteolysis of Yak Mozzarella Cheese During Ripening

**Figure 2:**
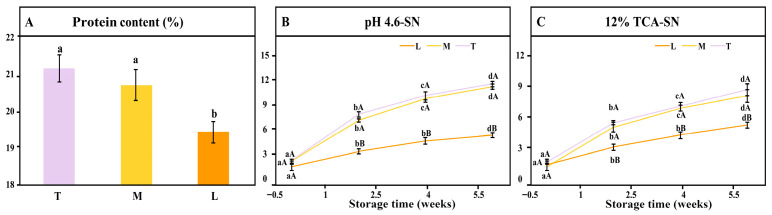
Changes in pH 4.6-SN, of 12% TCA-SN as a percentage of the total nitrogen during ripening ((**A**) protein content of yak mozzarella cheeses; (**B**) pH 4.6-SN as a percentage of the total nitrogen during ripening; (**C**) 12% TCA-SN as a percentage of the total nitrogen during ripening). Different lowercase letters indicate that the values of a parameter within the same group are significantly different after a specific storage duration, whereas different uppercase letters indicate that the values of a parameter within the same group are significantly different at the same storage time (*p* < 0.05). Error bars represent standard deviation.

Variations in acidification methods significantly affected the protein content (expressed on a dry-matter basis) of yak mozzarella cheeses (Figure 2). Compared with cheeses produced by culture acidification, L cheese showed significantly lower protein content, while M and T cheeses did not differ significantly, suggesting that subsequent proteolysis differences are mainly due to starter culture activity rather than initial protein composition.

Proteolysis plays a vital role in the development of cheese texture, functionality, and flavor [37]. As shown in Figure 2, extending ripening to 42 d significantly increased (*p* < 0.05) both pH 4.6-soluble nitrogen (pH 4.6-SN) and 12% trichloroacetic acid-soluble nitrogen (12% TCA-SN) in all cheeses, consistent with previous reports [22,38]. Proteolysis involves stepwise hydrolysis of casein into peptides and amino acids by residual rennet, starter proteases, and microbial enzymes [39,40]. pH 4.6-SN represents the overall extent of hydrolysis, while 12% TCA-SN reflects its depth by quantifying low-molecular-weight peptides and free amino acids.

The extent of proteolysis depends on milk enzymes, residual coagulant, starter activity, temperature, and pH [41]. Compared with directly acidified L cheese, T and M cheeses exhibited higher pH 4.6-SN values, exceeding those typically reported for cow mozzarella (~8–10% TN [42]). This difference may be linked to their lower ripening pH, which can help retain residual coagulant activity. Under acidic conditions, chymosin and pepsin are more stable and undergo slower thermal inactivation, especially when combined with the moderate temperatures used during stretching and subsequent storage [43]. Roughly 80% of starter proteases remain active after hot stretching and continue to hydrolyze casein [44]. Based on the biological characteristics of lactic acid bacteria, rod-shaped bacteria (e.g., *L. bulgaricus*) generally promote more extensive proteolysis than cocci (e.g., *L. cremoris* and *L. lactis*). Consequently, T cheese (containing both rod-shaped and coccal cultures) exhibited a slightly elevated pH 4.6-SN compared with M cheese (containing only cocci), even though their proteolysis rates during ripening were similar.

Secondary proteolysis, indicated by 12% TCA-SN, was significantly lower in L cheese than in T and M cheeses, reflecting the absence of active starter proteases and peptidases in direct acidification. Increasing the proportion of rod bacteria enhanced proteolytic depth, resulting in slightly higher 12% TCA-SN in T cheese than in M cheese-consistent with findings for mozzarella and other cheese types [45].

### 3.3. Cheese Functional Properties and Texture Profiles

#### 3.3.1. Hardness

**Figure 3:**
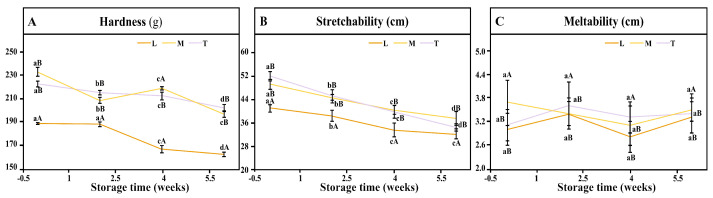
Textural characteristics of yak mozzarella cheese manufactured by different acidification methods with different ripening times ((**A**) hardness of mozzarella cheeses during ripening; (**B**) stretchability of mozzarella cheeses during ripening; (**C**) meltability of mozzarella cheeses during ripening). Different lowercase letters indicate significant differences across different storage periods within the same treatment group, whereas different uppercase letters indicate significant differences among different treatment groups at the same storage time (*p* < 0.05). Error bars represent standard deviation.

The unique texture of mozzarella cheese is primarily determined by the composition and structure of the rennet-induced curd. Overall, storage time significantly influenced texture profile analysis parameters, with the hardness of all samples decreasing steadily during ripening, indicating progressive softening (Figure 3A). This aligns with previous findings [12,32,46,47], which attributed softening to proteolysis and structural transformation from solid-like to more viscous states. The decline in TPA hardness paralleled the increase in pH 4.6-SN levels (Figure 3), supporting the link between proteolysis and texture degradation. Although direct-acidified cheese exhibited less proteolysis, its lower hardness was largely due to higher moisture content [48]. Inadequate whey drainage also led to higher water retention and an open, weaker curd structure [40]. Furthermore, hardness was positively correlated with total calcium content [49]. Differences in starter culture had no significant effect on TPA hardness, consistent with reports that rod-to-coccus ratios minimally affect cheese firmness [50].

#### 3.3.2. Stretchability and Meltability

Among the treatments, cheeses M and T showed similar stretchability, while L cheese was the lowest (*p* < 0.05) throughout ripening (Figure 3B). All yak mozzarella samples exhibited a progressive loss of stretch, with starter culture cheeses losing ~40% over 42 d compared with ~20% for the direct-acidified cheese. This reduction is attributed to proteolysis-induced breakdown of the casein network, release of colloidal calcium phosphate, and increased casein hydration [51]. Accordingly, M and T cheeses, which underwent more extensive proteolysis, showed greater loss of stretchability. Stretch behavior also depends on pH and calcium retention in the curd [52]. As pH moderately decreases, the solubility of calcium increases, which in turn weakens the calcium-bridge cross-linking between casein molecules. This structural change renders the casein network softer and more extensible [53]. Our previous study indicated that directly acidified cheeses retain more calcium than their culture-acidified counterparts. Consequently, the higher calcium retention associated with direct acidification may constrain the mobility and rearrangement capacity of the protein network, thereby imposing certain limitations on its stretchability.

Although meltability is also affected by proteolysis and the casein matrix, no significant differences were observed among cheeses during ripening (Figure 3C), consistent with Akarca [18]. Meltability is primarily governed by curd composition: lower moisture and excessive calcium both reduce melting [54]. Thus, L cheese showed reduced meltability compared to the other groups (Figure 3C).

### 3.4. VOC Profile of Yak Mozzarella Cheeses

The volatile organic compounds (VOCs) showing significant differences (*p* < 0.05) among the three yak mozzarella cheeses at different ripening periods (0, 14, 28, and 42 d) are listed in Table 1. Detected by SPME/GC-MS, these VOCs included ketones, aldehydes, alcohols, acids, and other minor compounds. Most VOCs identified here have also been reported in cow mozzarella [12,55] and originate from lipolysis, proteolysis, and catabolic reactions of amino acids, fatty acids, and residual carbohydrates. The biochemical pathways underlying VOC formation are complex and depend strongly on microbial enzymatic activity.

Ketones were the predominant class in all samples, consistent with previous studies [24,50]. Formed mainly through β-oxidation and decarboxylation of fatty acids, ketones contribute to the characteristic creamy aroma of cheese. Acetoin was the most abundant ketone in all yak mozzarella samples, with higher relative content than in cow mozzarella [56]. It imparts buttery and woody notes [50] and showed dynamic variation during ripening: initially higher in fermentation cheeses but declining sharply after 28 d, especially in M cheese. As a key metabolite of lactic acid bacteria, acetoin strongly reflects microbial activity [57,58]. Other VOCs were also more abundant in fresh M cheese than in T or L cheeses.

After 42 days of ripening, T and M cheeses (fermented with homofermentative starters) contained significantly more total alcohols (mainly ethanol and 2,3-butanediol) than directly acidified L cheese (*p* < 0.05). This distinction resulted from differing microbial interactions and metabolic pathways. In T and M cheeses, starter cultures collaborated with non-starter lactic acid bacteria (NSLAB): starter-generated intermediates such as pyruvate provided NSLAB with additional substrates and directed metabolic flow, while acetyl-CoA/diacetyl reduction boosted 2,3-butanediol production [59,60]. By contrast, alcohol formation in L cheese relied only on indigenous NSLAB and a limited number of adventitious microbes from raw milk, without the metabolic precursors or microbial cross-talk contributed by starters, leading to lower overall alcohol levels.

Acids also accumulated during ripening as a result of lipid and protein degradation. Aldehydes such as benzaldehyde and nonanal were transient intermediates that tended to be reduced to alcohols or oxidized to acids, affecting flavor balance [61].

**Figure 4:**
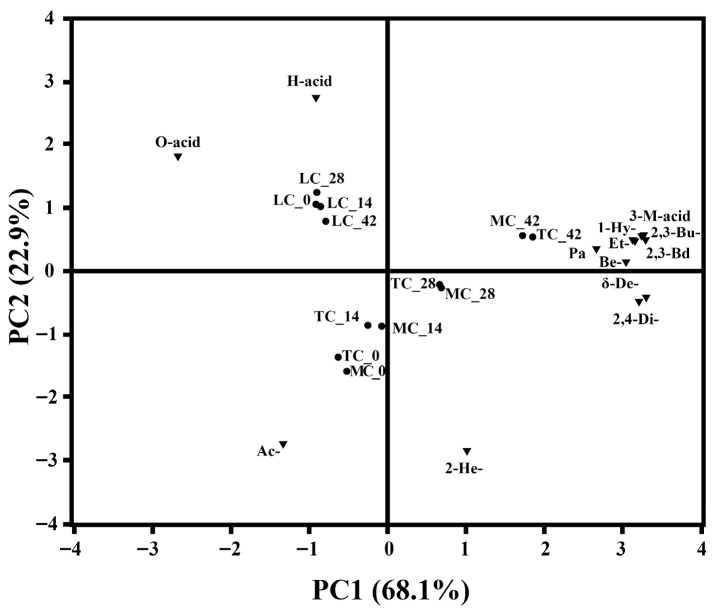
Two-dimensional PCA biplot (sample centroids and variable loadings) illustrating the volatile profile trajectories of yak Mozzarella cheeses during ripening. The analysis included all detected volatile organic compounds, whose concentrations were centered and scaled to unit variance before PCA. Variables with |loading| > 0.3 on either principal component are displayed and were considered major contributors to the observed separation.

Principal component analysis (PCA) was used to interpret VOC differences (Figure 4). PC1 and PC2 accounted for 68.1% and 22.9% of total variance, respectively. Fresh and ripened L cheeses clustered in the positive PC2 region, while T and M cheeses occupied the negative side, indicating clear separation by acidification method. Along PC2, T and M cheeses shifted from negative to positive regions over ripening, reflecting compositional evolution. Loadings revealed that acids such as hexanoic and octanoic acid contributed most to PC2, whereas acetoin-associated with fresh cheese aroma-loaded negatively, decreasing with aging. 2-Heptanone, derived from β-oxidation, was the major compound on the positive side of PC1. 2-Heptanone, generally, came from β-oxidation of saturated fatty acids load on the positive region of PC1.

To verify the endogenous origin of detected volatiles from cheese metabolism rather than external contamination, potential artifacts were evaluated. 2,4-di-tert-butylphenol, a typical plastic packaging migrant, was monitored as a marker. With analytical blanks and controls (unused packaging) included throughout the workflow, none of the key discriminatory compounds were detected in these samples, confirming their derivation from the cheese matrix itself.

### 3.5. Consumer Acceptance

Results of the consumer acceptance test for fresh and ripened yak mozzarella cheeses produced by direct acidification or culture acidification are presented in Table 2. The cheese made with a mesophilic multiple-strain starter and ripened for 42 d received the highest overall liking score, while both fresh and ripened L cheeses scored lowest. While texture undoubtedly influences acceptance, its impact in this context appears secondary. L cheese received relatively high texture liking scores yet achieved the lowest overall liking. This preference likely reflects consumers’ greater sensitivity to aroma and flavor, with texture also influencing acceptance. Similar findings were reported by Cais-Sokolińska [62], who noted that aroma was a key factor for young and middle-aged consumers. Consequently, ripened T cheese, which ranked second in aroma and flavor, achieved the second-highest overall liking score.

While differences in appearance and color were noted, overall liking and purchase intent tracked aroma and flavor preferences more strongly than visual characteristics [63]. According to JAR scores, L cheese was perceived as lacking flavor and creaminess, and being too light and soft. Overall, consumers favored the M cheese produced with mesophilic multiple-strain starter and expressed higher purchase intent for this variety.

## 4. Conclusions

Manufacturing yak mozzarella cheese using either starter cultures or direct acidification is feasible. Although the yield of L cheese (produced by direct acidification) was higher than that of T and M cheeses, consumers showed a clear preference for yak mozzarella made through culture acidification with a mesophilic multi-strain starter. The volatile organic compound data obtained in this study demonstrated that the contents of key flavor substances in T and M cheeses increased significantly after 42 d of storage. These compounds originated from distinct metabolic pathways: lipolysis (e.g., 2-heptanone), amino acid metabolism (e.g., benzaldehyde, 3-methylbutanoic acid), carbohydrate fermentation (e.g., acetaldehyde, 2,3-butanediol, ethanol), and lipid transformation (e.g., δ-decalactone). Furthermore, proteolytic activity influenced the textural and functional properties of the cheeses, such as meltability and stretchability, which also contributed to the differences in consumer acceptance among the samples. Overall, this study underscores the importance of volatile organic compounds and sensory attributes in determining consumer preference for yak mozzarella cheese.

Limitations and Future Research: This study has several limitations: (i) the limited number of independent manufacturing batches may constrain the generalizability of the statistical inferences; (ii) the study did not systematically evaluate microbial safety or shelf-life over the 42 d storage period; and (iii) the absence of measurements such as salt-in-moisture ratio and soluble/colloidal calcium distribution hindered a more mechanistic interpretation of texture and functional properties. Future work should include increased biological replication, systematic shelf-life assessments, and the application of molecular sensory techniques to further elucidate the formation and regulation of key flavor compounds.

## Figures and Tables

**Table 1:** Quantitative average amount (μg/kg) for VOC with statistically significant differences in yak mozzarella cheeses. Different lowercase letters indicate that the values of a parameter within the same sample are significantly different after a specific storage duration (*p* < 0.05).

Compounds	T Cheese				M Cheese				L Cheese			
	Ripening Time (d)				Ripening Time (d)				Ripening Time (d)			
	0	14	28	42	0	14	28	42	0	14	28	42
Ketones												
Acetoin	1357.95 ^a^	949.73 ^b^	485.49 ^c^	181.61 ^d^	1409.24 ^a^	886.78 ^b^	492.13 ^c^	165.34 ^d^	443.27 ^a^	361.57 ^b^	388.48 ^b^	292.28 ^c^
2,3-Butanediol	-	4.88 ^c^	18.65 ^b^	42.73 ^a^	-	6.08 ^c^	15.33 ^b^	35.61 ^a^	-	-	-	0.71 ^a^
2-Heptanone	3.66 ^a^	3.01 ^b^	2.88 ^b^	1.63 ^c^	4.04 ^a^	3.39 ^b^	3.01 ^c^	2.45 ^d^	0.83 ^a^	0.24 ^a^	-	-
Aldehydes												
Benzaldehyde	0.14 ^d^	0.86 ^c^	2.40 ^b^	4.61 ^a^	0.32 ^c^	0.69 ^b^	1.22 ^a^	1.98 ^a^	-	-	-	-
Alcohols												
1-Hydroxy-2-propanone	-	-	2.02 ^a^	2.65 ^a^	-	-	1.35 ^a^	1.97 ^a^	-	-	-	-
Ethanol	3.62 ^d^	18.09 ^c^	86.12 ^b^	235.49 ^a^	11.25 ^d^	41.63 ^c^	102.45 ^b^	258.33 ^a^	5.87 ^bc^	4.92 ^c^	6.13 ^b^	7.79 ^a^
Phenethyl alcohol	-	-	1.38 ^b^	3.25 ^a^	1.04 ^d^	2.96 ^c^	5.08 ^b^	8.14 ^a^	-	0.89 ^ab^	0.57 ^b^	1.05 ^a^
(R, R)-2,3-Butanediol	-	1.99 ^c^	5.31 ^b^	13.58 ^a^	-	3.54 ^c^	8.67 ^b^	22.15 ^a^	-	-	-	0.65 ^a^
Acids												
Hexanoic acid	4.21 ^c^	4.58 ^b^	4.86 ^ab^	5.02 ^a^	3.81 ^c^	4.42 ^b^	4.99 ^ab^	5.36 ^a^	7.06 ^c^	6.31 ^b^	6.98 ^bc^	5.16 ^a^
Octanoic acid	2.68 ^a^	1.89 ^b^	1.02 ^c^	0.22 ^d^	3.02 ^a^	2.12 ^b^	1.23 ^c^	0.34 ^d^	8.83 ^a^	8.37 ^a^	8.58 ^a^	8.21 ^a^
3-Methylbutanoic acid	-	8.46 ^c^	19.68 ^b^	38.14 ^a^	-	7.66 ^c^	15.20 ^b^	31.43 ^a^	2.34 ^a^	2.85 ^a^	2.18 ^a^	2.57 ^a^
Others												
δ-Decalactone	0.20 ^d^	0.47 ^c^	0.83 ^b^	1.21 ^a^	0.36 ^d^	0.52 ^c^	0.96 ^b^	1.34 ^a^	-	-	-	-
2, 4-Di-tert-butylphenol	0.19 ^d^	0.56 ^c^	0.97 ^b^	1.62 ^a^	0.34 ^c^	0.65 ^b^	0.88 ^ab^	1.01 ^a^	-	-	-	-

“-” denotes that the compound was not detected (ND; below the detection limit) at the corresponding time point; these data points were excluded from the ANOVA, and the significance was assigned based solely on quantifiable data to ensure methodological transparency and rigor.

**Table 2:** Overall liking scores in consumer acceptance testing of yak mozzarella cheese.

Item	T Cheese		M Cheese		L Cheese	
	Ripening Time (d)		Ripening Time (d)		Ripening Time (d)	
	0	42	0	42	0	42
Liking ^1^						
Appearance	5.7 ^c^	6.1 ^abc^	5.5 ^c^	6.2 ^ab^	6.7 ^a^	6.5 ^a^
Aroma	5.6 ^b^	6.4 ^a^	5.7 ^b^	6.8 ^a^	5.2 ^b^	5.4 ^b^
Color	6.2 ^b^	6.7 ^a^	6.3 ^ab^	6.6 ^ab^	6.1 ^b^	6.4 ^ab^
Flavor	5.2 ^ab^	5.7 ^a^	5.3 ^a^	5.9 ^a^	4.8 ^b^	5.2 ^ab^
Texture	4.9 ^b^	6.4 ^a^	5.2 ^b^	6.4 ^a^	6.2 ^a^	6.2 ^a^
Creaminess	5.3 ^b^	6.6 ^a^	5.7 ^ab^	6.4 ^a^	5.9 ^a^	6.1 ^a^
Overall	6.2 ^ab^	6.6 ^a^	6.1 ^b^	6.8 ^a^	5.6 ^c^	5.9 ^bc^
JAR questions ^2^						
Flavor (%)						
Not enough flavor	30.19 (16)	26.42 (14)	32.07 (17)	22.64 (12)	71.69 (38)	67.92 (36)
JAR	52.83 (28)	60.38 (32)	49.06 (26)	54.72 (29)	26.42 (14)	30.19 (16)
Too much flavor	16.98 (9)	13.20 (7)	18.87 (10)	22.64 (12)	1.89 (1)	1.89 (1)
Color (%)						
Too light	18.87 (10)	24.53 (13)	16.98 (9)	24.53 (13)	43.40 (23)	37.74 (20)
JAR	49.06 (26)	54.72 (29)	52.83 (28)	56.60 (30)	43.40 (23)	50.94 (27)
Too dark	32.07 (17)	20.75 (11)	30.19 (16)	18.87 (10)	13.20 (7)	11.32 (6)
Texture (%)						
Too soft	13.21 (7)	33.96 (18)	11.32 (6)	37.74 (20)	41.51 (22)	45.28 (24)
JAR	41.51 (22)	52.83 (28)	43.40 (23)	50.94 (27)	50.94 (27)	49.06 (26)
Too firm	45.28 (24)	13.21 (7)	45.28 (24)	11.32 (6)	7.55 (4)	5.66 (3)
Creaminess (%)						
Not creamy enough	39.62 (21)	28.30 (15)	43.40 (23)	26.42 (14)	41.51 (22)	37.74 (20)
JAR	37.74 (20)	43.40 (23)	35.85 (19)	47.16 (25)	43.40 (23)	45.28 (24)
Too creamy	22.64 (12)	28.30 (15)	20.75 (11)	26.42 (14)	15.09 (8)	16.98 (9)
Purchase intent ^3^	3.1 ^b^	3.7 ^a^	3.2 ^b^	4.1 ^a^	2.3 ^c^	2.6 ^c^

^a–^^c^ Means within a row with different superscripts are significantly different (*p* < 0.05). ^1^ A 9-point hedonic scale was used, where 1 = dislike extremely and 9 = like extremely. ^2^ The percentage of consumers who selected these options is presented, and numbers in parentheses represent the number of people selected. ^3^ A 5-point scale was used, and 1 = definitely would not buy, 2 = probably would not buy, 3 = may or may not buy, 4 = probably would buy, and 5 = definitely would buy.

## Data Availability

The original contributions presented in the study are included in the article, further inquiries can be directed to the corresponding authors.

## Abbreviations

The following abbreviations are used in this manuscript:

TS	Thermophilic multiple strain starter
MS	Mesophilic multiple strain starter
LA	Lactic acid
